# The Use of Unmanned Aerial Vehicles (UAV) on Delivering Biological Samples for COVID-19 and Tuberculosis Diagnosis: A Scoping Review

**DOI:** 10.1007/s43441-025-00865-0

**Published:** 2025-09-06

**Authors:** Olga Maíra Machado Rodrigues, Izabela Gimenes Lopes, Mariah Eduarda Ferreira de Oliveira, Mônica Angélica Carreira Fragoso, Maria Regina Fernandes de Oliveira, Raquel Santos Silva, Glaura Regina de Castro e Caldo Lima, Amílcar Sabino Damazo, Wagner de Jesus Martins

**Affiliations:** 1 Tropical Medicine Center, University of Brasilia, Brasilia, DF Brazil; 2 Fiocruz Brasilia, ColLaboratory of Science, Technology, Innovation and Society (CTIS), Brasilia, DF Brazil; 3 Public Management Innovation Laboratory, ENSP/Fiocruz), Escola Nacional de Saúde Pública Sérgio Arouca, Rio Janeiro City, RJ Brazil; 4 Institute of Biological Sciences, University of Brasilia, Brasilia, DF Brazil; 5 University Center of Planalto Central Aparecido dos Santos (UNICEPLAC), Brasilia, DF Brazil; 6 Institute for Health Technology Assessment (IATS), Federal University of Rio Grande do Sul (UFRGS), National Council for Scientific and Technological Development (CNPq), Porto Alegre, RS Brazil; 7 Laboratório Central de Saúde Pública do Distrito Federal, Secretaria de Estado da Saúde do Distrito Federal, Secretaria de Estado da Saúde do Distrito Federal, Brasilia, DF Brazil; 8 Fiocruz Brasilia, ColLaboratory of Science, Technology, Innovation and Society (CTIS), Avenida L3 Norte, s/n, Campus Universitário Darcy Ribeiro, Gleba A, Asa Norte, Brasilia, DF 70.904-130 Brazil

**Keywords:** Unmanned aerial vehicle, Delivery of health care, Transportation, Tuberculosis, COVID-19

## Abstract

**Purpose:**

To identify and review scientific evidence from experimental studies utilizing unmanned aerial vehicles (UAVs) to transport samples for the diagnosis of COVID-19 and tuberculosis (TB). This exploratory study aims to support the future development of UAVs for transporting biological samples within the Brazilian Unified Health System (SUS).

**Methods:**

This scoping review defined its eligibility criteria using the PECO acronym, focusing on: **P**opulation: biological samples for diagnosing COVID-19 or TB; **E**xposure: UAV transportation; **C**omparator: land transportation; **O**utcomes: Cost, effectiveness, methods for sample preservation, flight parameters (time, altitude, speed, distance), and quality of transported samples. Eligible studies were identified through searches in Medline via PubMed, Scopus, Embase, and Web of Science. Grey literature was explored via Google Scholar.

**Results:**

Of the 2,052 articles initially found, 797 were duplicates, 1,247 were screened by title and abstract and excluded, eight were retrieved (and fully read) of which five met the eligibility criteria and were included in the review. These studies provided diverse evidence regarding cost, operational performance, safety, and sample integrity.

**Conclusion:**

The reviewed studies demonstrate promising applications of UAVs in healthcare logistics. However, regulatory and legal frameworks require adaptation to ensure operational safety. Further experimental studies are necessary, particularly involving beyond visual line of sight (BVLOS) operations, to evaluate scalability and potential cost reductions.

**Supplementary Information:**

The online version contains supplementary material available at 10.1007/s43441-025-00865-0.

## Introduction

In a world marked by social inequalities and uneven access to goods, services, and new technologies, populations in remote and hard-to-reach areas often face delays in disease diagnosis due to logistical challenges in delivering samples to laboratories [1, 2]. Among the potential solutions for this reality, using unmanned aerial vehicles (UAVs), commonly known as drones, for transporting laboratory samples has been evaluated in various scenarios worldwide [1–6].

Initially developed for military purposes, UAVs have found applications in many other fields, such as mining, environmental assessment, agriculture, and healthcare, with their use being explored and tested worldwide [7]. In medical logistics, they can be used to transport low-weight, time-critical items, such as biological samples (blood, feces, urine, etc.), personal protective equipment, vaccines, medicines, and blood products [2, 7].

UAV flights have become safer in recent years, as several optimized functions, systems and computational resources have been developed to enable enhanced tracking and real-time monitoring of various parameters (including temperature of packages), detect and avoid obstacles and other aircraft, besides ensuring secure deliveries through data encryption [2, 8, 9].

The present study aims to identify and review scientific evidence from experimental studies on transportation of respiratory biological samples by UAVs, focusing on COVID-19 and tuberculosis (TB) diagnosing, by an exploratory design. The choice of these diseases was due to their Public Health magnitude in Brazil and because both base the diagnosis on low weighted respiratory samples (sputum for TB – approximately 3 to 5 ml; nasopharyngeal or oropharyngeal swab for COVID-19 – around 10 g including plastic tubes and 1 to 3 ml of viral transport medium) [10, 11].

Brazil is a country of continental proportions, marked by a vast diversity of socioeconomic and cultural realities that are also reflected in the health conditions, as well as in the local healthcare networks of its Unified Health System (SUS) [12]. With a population exceeding 215 million people, the country is divided into 5,570 municipalities spread across a territory of 8,510,000 km² [13]. The distances between municipalities can be immense. For example, the journey from São Gabriel da Cachoeira to Manaus, the capital of the state of Amazonas, spans approximately 850 km and takes around 24 h by express boat [14].

Regarding sample collection, the Brazilian Ministry of Health recommends a decentralized approach, prioritizing Primary Health Care as the preferred entry point within the SUS. Currently, there are 44,938 Basic Health Units (UBS) registered by the national SUS databases [15]. In addition to these primary care facilities, other health services – as hospitals and Emergency Care Units – can collect samples and forward them to laboratories capable of performing the necessary diagnostic tests.

The National Public Health Laboratory System (SISLAB) is hierarchically structured and operates across federal, state, and municipal levels, in accordance with the SUS principles. It encompasses national laboratory networks dedicated to epidemiological, environmental and sanitary surveillance, besides high-complexity medical care. The system includes a range of facilities, such as local laboratories, border laboratories, municipal, state, and national reference laboratories, as well as collaborating centers [16]. 

The findings of this review may support the development of UAVs for transporting biological samples within the SUS, improving diagnosis and treatment opportunities for diseases such as TB and COVID-19, particularly crucial for populations in remote and isolated areas, such as those living in the Amazon Region.

As of this article’s publication, no other review found has specifically addressed this topic.

## Materials and methods

### Study Design

This is a scoping review, defined as a type of evidence synthesis that systematically identifies and maps the breadth and nature of existing research on a given topic. It includes studies from various sources and contexts, aiming to describe the main characteristics, themes, and knowledge gaps related to the subject [17]. 

### Protocol and Registration

The review was conducted in accordance with the recommendations of the Preferred Reporting Items for Systematic Reviews and Meta-Analyses for Scoping Reviews (PRISMA-ScR) extension (Supplementary Table 1), which consists of a 22-item checklist with recommended sections and subsections for scoping reviews [18]. The registered study protocol is available at the Open Science Framework Database (< 10.17605/OSF.IO/K6RHS>), on the link: <https://osf.io/k6rhs>.

### Eligibility Criteria

The review sought to answer the question: “What is the state of the art regarding the transportation of TB and COVID-19 biological samples by UAV?“, using the population/exposures/comparisons/outcomes (PECO) framework for its definition, aiming to fill the identified knowledge gaps, as described below:

- **P**: biological samples for COVID-19 or TB diagnosis;
- **E**: UAV transportation;
- **C**: land transport or absent comparator;
- **O**: cost assessment, effectiveness, sample preserving methods during flights, time saved, altitudes, distances, speeds reached by the UAV and quality assessment of the transported samples.

The eligibility criteria that guided the process of selecting studies were based on the same PECO framework. The inclusion criteria comprised primary experimental studies examining the use of UAVs for transporting samples related to TB or COVID-19 diagnosis.

The exclusion criteria included case studies, reviews, observational studies, letters to editors, editorials, comments, conference abstracts, book chapters, and articles that deal only with theoretical modeling.

Interest variables were cost assessment, effectiveness, time, altitudes, distances and speeds reached, sample preserving methods during flights, and quality of the samples transported by UAV.

#### Definitions

- UAV: powered, aerial vehicles that do not carry a human operator, use aerodynamic forces to generate lift, may fly autonomously or be remotely piloted, and can be expendable or recoverable, carrying either lethal or nonlethal payloads, as defined by the Medical Subject Headings (MeSH) [19].
- Pulmonary TB samples for diagnosis: sputum samples.
- COVID-19 samples for diagnosis: nasopharyngeal or oropharyngeal swabs or simulated samples.

### Information Sources

The electronic search for scientific evidence used a structured search strategy (see Sect. 2.5.), carried out until January 31, 2025, in the following databases: PubMed (< http://www.ncbi.nlm.nih.gov/sites/pubmed>); Scopus (< http://www.scopus.com>); Embase (< https://www.embase.com>), accessed through Elsevier (< https://www.elsevier.com>); Web of Science – WOS (< https://www.webofknowledge.com>), accessed through Clarivate Analytics (< https://clarivate.com>). Additionally, Google Scholar (< https://scholar.google.com>) was consulted for grey literature.

### Search Strategy

The structured PECO-based search strategy was developed for MEDLINE (via PubMed) using Boolean operators (AND/OR), incorporating Medical Subject Headings - MeSH (available at < https://www.ncbi.nlm.nih.gov/mesh/advanced>), as well as input terms, free-text terms and keywords also combined by Boolean AND/OR operators (Table 1).

**Table 1:** Electronic database search

Data source	Search string
MEDLINE (via PubMed)	*((Sputum[mesh] OR Sputums[tiab] OR Sputums*,* Induced[tiab] OR COVID-19 [mesh] OR COVID-19 [tiab] OR 2019 nCoV Infection[tiab] OR SARS-CoV-2 Infection[tiab] OR SARS-CoV-2 Infections[tiab] OR 2019 Novel Coronavirus Infection[tiab] OR COVID 19 Virus Infection[tiab] OR Coronavirus Disease-19[tiab] OR Severe Acute Respiratory Syndrome[tiab] OR Tuberculosis[mesh] OR Tuberculosis[tiab] OR Kochs Disease[tiab])) AND ((Unmanned Aerial Devices[mesh] OR Transportation[mesh] OR Unmanned Aerial Devices[tiab] OR Transportation[tiab] OR Aerial Device*, Unmanned[tiab] OR Drone*[tiab] OR Vehicle*, Unmanned Aerial[tiab])) AND ((Costs and Cost Analysis[mesh] OR Specimen Handling[mesh] OR Costs and Cost Analysis[tiab] OR Specimen Handling[tiab] OR Affordability[tiab] OR Pricing[tiab] OR Cost*[tiab] OR Cost Measure*[tiab] OR Handling, Specimen*[tiab] OR Specimen Collection*[tiab] OR Collection, Specimen*[tiab]))

The search string was adapted for each database according to its specific syntax requirements. For Google Scholar, the same strategy was altered. The initial selection for Google Scholar title reading comprised the first 201 matches, processed manually to check whether potential eligible studies were missing from the primary database search. Duplicates across databases were identified and removed to ensure each record was counted only once.

### Data Screening and Extraction

The retrieved documents were exported and organized using the reference management software Rayyan™ Web and Mobile App for Systematic Reviews (available at < https://www.rayyan.ai/>). Duplicates records were manually removed and verified by two reviewers (O.M.M.R and M.E.F.O). Initial screening of titles and abstracts was conducted by one reviewer (O.M.M.R.), a health professional with expertise in systematic review methodology. Studies excluded during this phase were re-evaluated by a second reviewer (I.G.L.), also a health professional experienced in systematic review procedure. The screening process utilized Rayyan^®^’s blinded review functionality to ensure unbiased selection according to the PECO framework.

Full-text articles were independently assessed for eligibility by two reviewers (O.M.M.R. and I.G.L.). Disagreements were resolved through consensus meetings with a third senior reviewer (M.A.C.F.). Reasons for exclusion were recorded after examining the full text. Articles published in languages not understood by the reviewers were translated using the Google Translate tool (available at < https://translate.google.com>).

Three reviewers (O.M.M.R.; M.A.C.F. and I.G.L.) independently conducted manual data extraction.

The studies’ primary characteristics, including the first author, year of publication, country of research, study design, sample sort, UAV model, comparator (if any), UAV operation; quantitative parameters (number of samples transported per route by UAV, per-sample costs; average and maximum speeds; maximum altitude reached by the UAV; minimum and maximum temperatures in °C during transport; number and percentage of successful routes without sample losses); and qualitative parameters (e.g. care for preserving samples; sample quality assessment after transport) were identified and organized in Excel spreadsheets (Excel^®^, Microsoft^®^, USA).

### Data Synthesis and Analysis

Characteristics of interest from the included studies were summarized and tabulated in spreadsheets using Excel software (Excel^®^, Microsoft, USA). Qualitative and quantitative data were extracted and synthesized in descriptive tables. Qualitative data were analyzed thematically and integrated narratively to provide a comprehensive synthesis of reported findings.

## Results

### Selected Articles

**Fig. 1:**
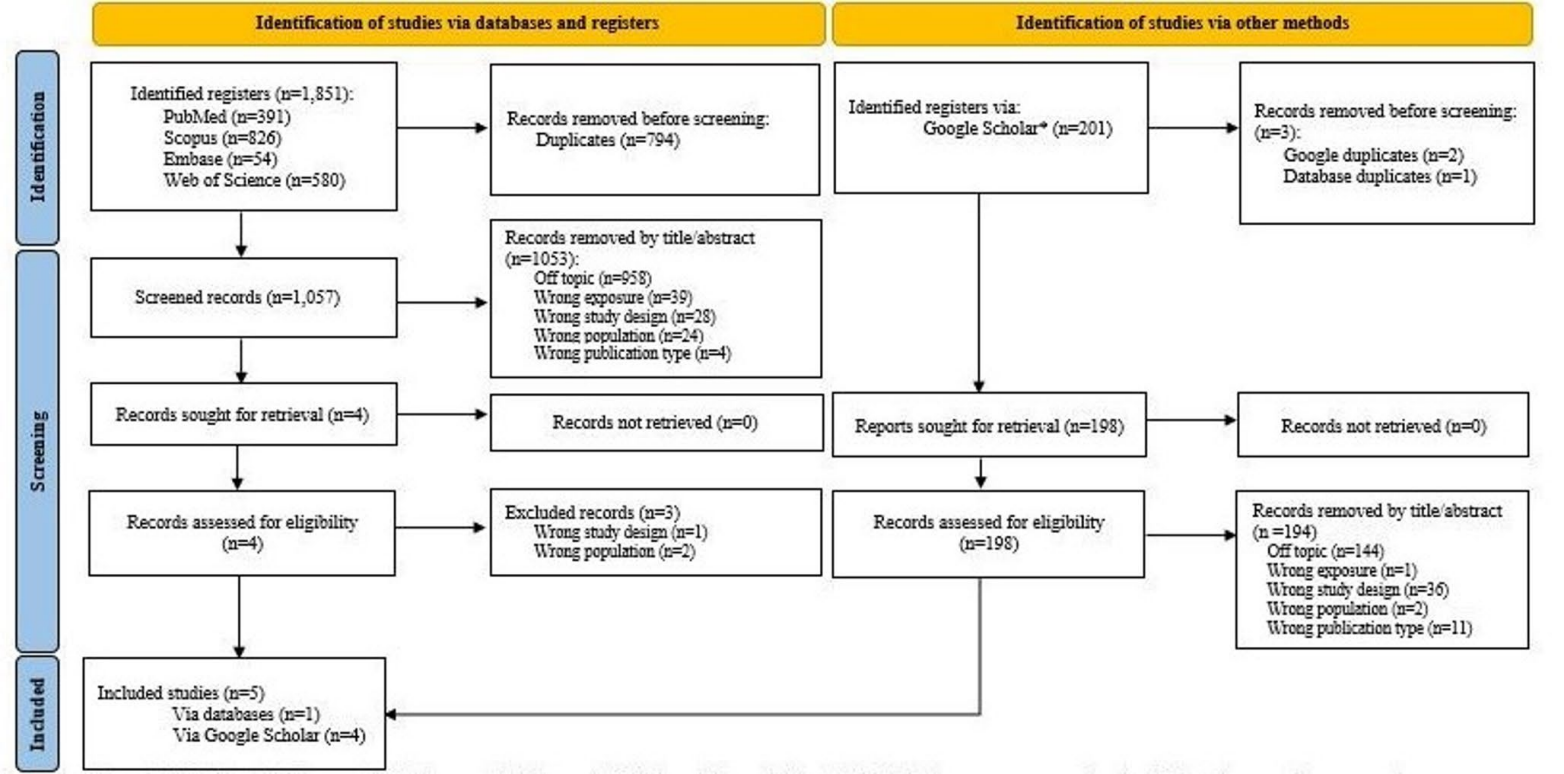
PRISMA 2020 flow diagram for new systematic reviews which included searches of databases, registers and other sources.^20^ *Reference*: Page MJ, McKenzie JE, Bossuyt PM, Boutron I, Hoffmann TC, Mulrow CD, et al. The PRISMA 2020 statement: an updated guideline for reporting systematic reviews. BMJ. 2021; 10.1136/bmj.n71

Of the 2,052 articles initially found, 797 were duplicates, 1,247 were screened by title and abstract and excluded, eight were retrieved (and fully read) of which five met the eligibility criteria and were included in the review. Figure 1 (PRISMA 2020 flow diagram for new systematic reviews) summarizes the identification, screening, and inclusion of articles [20]. Table 2 presents the characteristics of the selected studies, including author, year, country, sample sort, UAV model, comparator, and UAV operation.

**Table 2:** Summary of selected studies’ characteristics (author, year, country, sample sort, UAV model, comparator, and UAV operation)

Author (Year), country	Sample sort	UAV model	Comparator (if any)	UAV operation
García et al. (2021), Spain	COVID-19 vials simulating samples	DJI Matrice 300 RTK (8 Km)	Absent	VLOS*, EVLOS** and BVLOS***
Flemons et al. (2022), Canada	COVID-19 simulated samples	SwissDrone SDO 50 V3 (300 Km)	Stationary samples	VLOS*, EVLOS** and BVLOS***
Malamule et al. (2022), Mozambique	Sputum for TB diagnosis	Swoop Aero Kookaburra Aircraft (135 Km)	Delivery by a car	-
Sylverken et al. (2022), Ghana	Naso or oropharyngeal swabs for COVID-19 diagnosis	Zipline drone	Absent	-
Thakur et al. (2022), India	Sputum for TB diagnosis	-	Delivery by motorbikes	VLOS*

- No data

*VLOS - Visual Line of Sight

**EVLOS - Extended Visual Line of Sight

***BVLOS - Beyond Visual Line of Sight

### UAV Payload Capacity and Safety

Among studies that reported UAV payload specifications, weight capacity ranged from 3 to 45 kg [1, 2, 21], and the volume from 1,200 to 5,362.5 cm^3^ [1, 21]. The number of samples transported per UAV flight varied from 4 to 50 per route [1, 2, 8, 22]. 

Reported flight safety concerns included unsafe routes, weather conditions and UAV loss of C2 link (“Command and Control” link, which is the communication pathway between the UAV and its ground control station) which can lead to crashes [2, 8, 21, 22].

Suggested solutions comprised defining safe routes [2, 21], adhering to weather conditions protocols [2, 8, 21, 22], using UAV safety devices (e.g., parachutes) and appropriate containers [1, 2, 8, 21], and complying with national Aviation Regulations for UAV transport [2, 21]. One article emphasized the need to develop a C2 link based on a redundant navigation system (e.g., radio frequency and cellular network) [21], and other cited the importance of community awareness campaign prior to the drone flights, ensuring that the population is informed about the purpose, procedures, and protocols for potential adverse events [1].

About the success of UAV flights, García et al. reported that 10 out of 16 flights happened without unexpected events (success rate of 62.5%) [21]. Thakur et al. successfully completed 151 of 180 planned UAV flights (83.8%). Other studies did not report losses. Mentioned causes of UAV operations failures encompassed low flight altitude, coordinate air traffic control (ATC) failure, unsynchronized ground control station, loss of C2 link, balloon trajectory flight, and weather conditions [21, 22].

Table 3 summarizes the characteristics of the UAV used in the included studies, focusing on payload capacity and safety of the flights.

**Table 3:** Summary of UAV payload capacity and flights safety

Author (Year), country	UAV max. payload capacity		Total number of samples transported	Average number of samples transported per route	Total flights performed	Successful flights*	
	Kg	cm^3^				*n*	Fraction (%)
García et al. (2021), Spain	8	3,769.9	-	-	16	10	10/16 (62.5)
Flemons et al. (2022), Canada	45	-	10	10	2	2	2/2 (100,0)
Malamule et al. (2022), Mozambique	3	5,362.5	156	8,8	18	18	18/18 (100,0)
Sylverken et al. (2022), Ghana	-	-	2,537	5,8**	440	440	440/440 (100,0)
Thakur et al. (2022), India	-	-	351	4	151	151	151/180 (83.8)

- No data

* Successful flights transporting COVID-19 or TB samples, in relation to the ones planned, without citing unexpected events or losses

** Arithmetic mean, obtained from the absolute values presented in the article (2,537 samples in 440 flights)

### Samples Conservation

To ensure sample conservation, the studies adhered to standard collection and packaging protocols, including triple packaging in accordance with IATA (International Air Transport Association) and WHO (World Health Organization) guidelines, as well as the use of ice packs and temperature monitoring sensors [1, 2, 8]. Encoding sample identification (e.g., barcoded) was a strategy used to maintain patient confidentiality and sending case investigation forms [1, 8].

Two studies reported temperature monitoring of cargo box (or container) during the flights. In the first, it ranged from 3 °C to 8 °C during air transportation, while control samples ranged from 2 °C to 6 °C during ground transportation [1]. In the second, the UAV container carried individually compartmentalized supplies with refrigeration maintained at 4 °C [2].

### Distance Travelled, time Saved and Flight Parameters

Analysis of UAV flight distances was limited, as the included studies reported only land travel distances rather than precise aerial route measurements.

Sylverken et al., in Ghana, reported that the average travel time by road from the Zipline office to the Kumasi Centre for Collaborative Research (KCCR) was 96 min over a distance of approximately 63 km. The same route covered by an UAV took an average of 39 min, with a range of 36.5 to 43.3 min. However, the study did not provide a precise measurement of the actual distance traveled by the UAV between the Zipline office and KCCR [8]. 

Thakur et al., in India, found that UAVs reduced delivery time by approximately threefold, with an average duration of 7.1 ± 0.8 min compared to 22.7 ± 4.6 min by motorbike. The study reported a clear difference in the mean distances traveled: 12.09 ± 1.6 km by motorbike *versus* 2.89 ± 0.35 km by UAV [22]. 

The longest time of flight registered was 42 min [2]; flight altitude ranged from 40 to 90 m [2, 21]; and the average flight speed reported from 10 to 93 km/h [1, 2] (Table 4).

**Table 4:** UAV flights comparable parameters: maximum distance travelled per charge, altitude, time of flight and average speed

Author (Year), country	Max. distance travelled per charge (Km)	Max. altitude (meters)	Time of flight (min.)		UAV average speed (Km/h)
			Max.	Average	
García et al. (2021), Spain	8	90	-	-	-
Flemons et al. (2022), Canada	300	40	42	-	10
Malamule et al. (2022), Mozambique	135	-	90*	25	93
Sylverken et al. (2022), Ghana	-	-	-	39	-
Thakur et al. (2022), India	-	-	-	7,1	-

- No data available

* Aircraft battery endurance per charge of the UAV model used

** Estimation based on the data available on the article. For total duration in min (mean ± SD) = 7.1 ± 0.8

### Cost and Effectiveness

Flemmons et al., in Canada, provided a broader overview of UAV acquisition costs, highlighting significant cost variability, depending on the expected resistance of the UAV model: medium resistance ones range between US$ 7,000 and US$ 15,000, while high resistance ones can cost from US$ 200,000 to US$ 600,000 [2].

Thakur et al. assessed per route costs and concluded that each UAV trip (US$ 0.30) was more than four times cheaper than the motorcycle route (US$ 1.30) [22].

### Quality Assessment of Transported Samples

Two studies evaluated the quality of the biological respiratory samples transported by UAV. One of them did not identify any changes compared to land transport, founding fungal contamination in TB respiratory samples in solid culture at a rate of 3% (five samples) for UAV and 1% (two samples) for land transport, with no contamination in the liquid culture [1]. The other, which tested the reliability of the COVID-19 test and spiked viral transport media or saline used in COVID-19 or other respiratory virus sampling kits under varying temperatures and higher altitudes, considering the conditions at the simulation site, did not get sufficient data to validate the proof-of-concept, and recommended further research to access how extreme environmental and weather conditions may affect the flights performance and specimen integrity [2].

### Regulatory and Legal Landscape

The regulatory and legal landscape were cited by Flemons et al. as one of the primary challenges in establishing drone-based delivery for healthcare and other purposes in Canada. In this context, developing tested and approved Standard Operating Procedure (SOP) and safety documents was defined as a critical prerequisite for enabling sector-wide innovation and impact [2]. 

García et al., in Spain, highlighted that, under European Union (EU) regulatory framework, delivery operations required extended range fall under specific regulations due to their interest in operating beyond visual line of sight (BVLOS) and extended visual line of sight (EVLOS). BVLOS operations rely on detect and avoid (DAA) systems to ensure collision prevention. Approval for BVLOS operations also requires a Specific Operations Risk Assessment (SORA) and authorization. To obtain authorization for the operations by AESA (Spanish Safety Aviation Agency), it was necessary to assess risk performing a SORA for each of the three scenarios tested. Moreover, for “dangerous goods” like medical items (e.g., pathogen samples or untested blood), additional safety measures, such as the use of secure container, are mandated to prevent risks during transport [21]. 

Flemons et al. worked with Transport Canada to obtain approval for BVLOS operations based on the robust standard operating procedures, safety and emergency procedures, besides developing detect and avoid (DAA) systems by the project. In terms of safety documentation, SOPs for each UAV, payload system, and flight operation tested (VLOS, EVLOS and BVLOS), were enhanced to include checklists aligned with the Canadian Aviation Regulations (i.e., site survey, preflight, and postflight), and mission mapping and safety details [2]. 

With regard to transporting infectious substances (i.e., diagnostic samples), transportation requirements (governed by Transport Canada and the United Nations – UN – designations for the transport of dangerous goods) will need to be updated and amended to include drones [2]. 

## Discussion

The main findings of this review are: (1) limited experimental studies exists on the use of UAVs to deliver respiratory samples for TB and COVID-19 diagnosis; (2) payload capacities, distances traveled, and associated costs vary widely depending on the UAV model; (3) key safety concerns include unsafe routes, weather conditions and UAV loss of C2 link which may result in crashes (with consequent risks of accidents, pathogen spread and sample loss); (4) proposed risk mitigation strategies include defining safe routes, respecting weather condition, using UAV safety devices (e.g., parachutes) and proper containers, adhering to national Regulations for UAV transports, developing navigating systems based on a redundant communication network, and conducting community awareness campaigns prior to the UAVs flights; (5) sample quality depends the adherence to pre-analytical phase protocols and the temperature maintenance during the flights; (6) compliance with aviation legal and regulatory frameworks maybe is the major challenge to UAV-based delivery of biological samples [1, 2, 8, 21, 22]. 

Although several scientific articles have addressed the transport of biological samples since 2016, when Amukele and colleagues [23], in the USA, demonstrated that UAVs could be suitably used for transportation of laboratory specimens, few experimental studies outside Africa have specifically focused on a UAV-based delivery of respiratory samples for diagnosis of COVID-19 or TB. As pointed for some authors, it is necessary to develop more studies on the quality of the samples transported by UAV in different environmental conditions, in order to increase the confidence in this kind of delivery [1, 2, 24, 25]. In this sense, it is important to notice that the in African continent, at least other three countries (Madagascar, Malawi and Senegal) have reported experiences using UAVs for sample delivery, two of them aiming to improve the diagnosis and treatment of TB through this technology [24]. Articles related to these experimental efforts were not identified in the present review.

Even though this review suggests the potential cost-effectiveness of UAV-based sample transport, scientific evidence on this aspect remains inconclusive. Different settings may influence the economics of UAV services in healthcare [3]. While a study in Norway estimated that UAV transport could theoretically save NOK 100 million (EUR 9.5 million) per year [26], a data modeling study in the United Kingdom (UK) suggested it could increase overall costs by 56% – excluding expenses related to airspace management and dangerous goods training [25].

To better evaluate the impacts, future studies should incorporate standardized indicators for a UAV-supported healthcare system, including costs, time savings, and CO^2^ emission factors [24, 25]. These metrics can provide a comprehensive assessment framework and support informed decision-making.

Enhancing the safety of UAV flights begins with selecting a UAV model aligned with the operational requirements, including the distance to be traveled, payload capacity, and the environmental conditions of the target location. Ensuring safety involves mapping secure routes and adherence to weather conditions, alongside the using specialized UAV containers and built-in safety features such as parachutes and flotation systems for operations over water. Furthermore, the integration of detect-and-avoid systems, as well as redundant navigation systems combining radio frequency, cellular networks, and satellite communication, significantly enhances operational reliability and flight safety. Additionally, population awareness campaigns regarding UAV flights should also be considered to foster community acceptance and minimize resistance [1, 2, 21, 25].

To ensure the quality of transported samples, comprehensive and rapid training is essential. This includes SOP for the collection of diagnostic samples for TB and COVID-19, as well as providing specialized training on the appropriate packaging and storage of these samples within UAV containers to preserve their integrity during transportation [1, 2, 8, 24]. It may also be desirable to develop dedicated container for the transport of biological samples for COVID-19 and TB (and other similar samples) with cooling and real-time temperature monitoring systems, and blockchain integration for secure data traceability.

Adhering to aviation legal and regulatory frameworks poses significant challenges for implementing UAV-based delivery of biological samples in Brazil. However, in February 2024, the Brazilian National Civil Aviation Agency (ANAC) took a groundbreaking step by granting, for the first time, an Approval of Exemption from Article E94.103(a) [27]. This certification allows a company to manufacture and operate UAVs for transportation of Category B Biological Substances (UN 3373) [27], which includes TB and COVID-19 patient samples, but not culture of these pathogens, what would require Category A Infectious Substance Affecting Humans (UN 2814) [28]. Nevertheless, this milestone sets a precedent that may pave the way for further initiatives, especially in support of the Brazilian Public Health System.

Regarding potential UAV routes in isolated regions of Brazil, and drawing on the studies reviewed - particularly the experience of Sylverken in Ghana [8] – it may be important to assess the feasibility of implementing strategic stopovers in route to the final delivery destinations at designated laboratories.

A limitation of this review is that it reflects the perspective of healthcare professionals rather than UAV engineering experts. While the team recognizes the urgent need to improve logistics in public healthcare, technical gaps may remain regarding UAV systems and operations. Additional limitations include the small number of studies meeting the review’s inclusion criteria, the lack of standardized indicators for UAV-supported healthcare systems, and the scarcity of studies examining the quality of samples transported by UAVs.

## Conclusion

This scoping review identified key considerations for acquiring or developing a UAV capable of transporting biological samples for TB and COVID-19 diagnosis within the Brazilian Unified Health System. Although the topic presents multiple complexities, the findings offer a valuable foundation to guide future initiatives in UAV-based sample logistics and diagnosis. Regulatory and legal frameworks require adaptation to ensure operational safety. Further experimental studies are necessary, particularly involving beyond visual line of sight (BVLOS) operations, to evaluate scalability and potential cost reductions.

## Supplementary Information

Below is the link to the electronic supplementary material.

Supplementary Material 1

## Data Availability

The review was conducted in accordance with the recommendations of the Preferred Reporting Items for Systematic Reviews and Meta-Analyses for Scoping Reviews (PRISMA-ScR) extension (Supplementary Table 1) and the registered study protocol is available at the Open Science Framework Database (< https://doi.org/10.17605/OSF.IO/K6RHS>), on the link: < https://osf.io/k6rhs>.
